# Unilateral Headache as a Presentation of Spontaneous Internal Carotid Artery Dissection: A Case Report

**DOI:** 10.7759/cureus.116408

**Published:** 2026-09-17

**Authors:** João Miguel F. Santos, Rosária C Leal, Joana Baptista, José Miguel Mendes

**Affiliations:** 1 Family Medicine, Unidade de Saúde Familiar (USF) Terras de Ferreira, Unidade Local de Saúde (ULS) Tâmega e Sousa, Paços de Ferreira, PRT

**Keywords:** cranial nerve palsy, general practice, internal carotid artery dissection, primary health care, pseudoaneurysm, ptosis, unilateral headache

## Abstract

Spontaneous internal carotid artery (ICA) dissection is a rare but potentially serious cause of ischemic stroke, with a frequently heterogeneous clinical presentation that poses a diagnostic challenge, particularly in primary care settings. We report a case of a 46-year-old female with a history of migraine, resolved thyrotoxicosis, and oral contraceptive use, who presented to her family health unit with a 15-day history of left-sided hemicranial headache preceded by ipsilateral neck pain without identifiable trauma. Associated findings included left ptosis, ipsilateral scalp hyperesthesia, and taste disturbance affecting the posterior third of the tongue, in the absence of motor deficits, aphasia, or visual changes. She was referred to the emergency department, where CT angiography revealed narrowing of the left ICA at the post-bulbar segment, raising suspicion of dissection, subsequently confirmed by cervical Doppler ultrasound and magnetic resonance angiography, which demonstrated a minimally opacified false lumen with mild-to-moderate stenosis and no cerebral ischemia. An extended etiological workup was negative. Dual antiplatelet therapy was initiated, and the patient achieved complete neurological recovery, being discharged on hospital day 7. Follow-up imaging identified the development of a pseudoaneurysm at the post-bulbar segment of the left ICA without hemodynamic significance. The patient remains under neurology and family medicine follow-up with persistent headache and cervicalgia under pharmacological management. This case highlights the importance of maintaining a high index of clinical suspicion in primary care when faced with persistent unilateral headache associated with cranial nerve signs, even in the absence of overt stroke, and underscores the role of the general practitioner in the early recognition of warning signs, timely referral, and continuity of care.

## Introduction

Internal carotid artery (ICA) dissection is a rare but clinically relevant cause of ischemic stroke, accounting for 2% to 2.5% of all ischemic strokes in the general population and for a substantially higher proportion in patients under 45 years of age, estimated at 10% to 25% of ischemic strokes in this age group [1]. The annual incidence ranges between 2.5 and 3 cases per 100,000 inhabitants, with a peak between the third and fifth decades of life [1].

Spontaneous ICA dissection results from a tear in the arterial intima, with consequent intramural hematoma formation that may cause luminal stenosis or occlusion, local thrombosis, and distal embolization. The etiopathogenesis remains incompletely understood. Implicated factors include genetic predisposition, notably fibromuscular dysplasia and connective tissue disorders, as well as mechanical triggers such as sudden or forced neck movements [2,3].

The clinical presentation is heterogeneous and frequently non-specific, contributing to diagnostic delay. The classic triad of ipsilateral neck and cranial pain, Horner syndrome, and focal neurological deficits secondary to cerebral ischemia is present in its complete form in fewer than one-third of cases [1]. Additional findings may include lower cranial nerve palsies, pulsatile tinnitus, and facial sensory disturbances, all of which may be overlooked without a high degree of clinical suspicion [4].

Head or neck pain is the inaugural symptom in more than half of patients with non-traumatic carotid artery dissection and may precede ischemic complications by days to weeks [5]. Recognition at this stage is challenging. In a multicenter international analysis of over 4000 patients with cervical artery dissection, approximately one in six had a medical encounter for attributable symptoms in the preceding 30 days without the diagnosis being made, and a third of these were reported to have had an ischemic stroke between that encounter and the eventual diagnosis. Younger age, a history of migraine, and presentation with headache and nonfocal symptoms were independently associated with delayed recognition [6].

We report a case of a 46-year-old female registered at Unidade de Saúde Familiar (USF) Terras de Ferreira, Unidade Local de Saúde (ULS) Tâmega e Sousa, Portugal, in whom a diagnosis of spontaneous left ICA dissection was established following referral by the general practitioner, with a favorable clinical outcome but complicated by pseudoaneurysm development on follow-up imaging.

## Case presentation

Patient information

The patient was a 46-year-old female photographer, residing within the catchment area of USF Terras de Ferreira, ULS Tâmega e Sousa, Portugal. She lived with her husband and two children. No family history of early-onset stroke, connective tissue disease, or arterial dissection was known.

Relevant past medical history included the following: migraine (managed with topiramate 50 mg twice daily and zolmitriptan 5 mg as needed); resolved thyrotoxicosis (no current medication); overweight (weight 78 kg, height 162 cm, BMI 29.7 kg/m2); three pregnancies, two deliveries, and one spontaneous miscarriage; prior tubal ligation. In February 2025, a desogestrel-only oral contraceptive (Azalia) had been initiated for menorrhagia associated with iron deficiency anemia, in the context of a uterine polyp/adenomyoma of approximately 8 mm under gynecological review. Primary care records documented previous high-normal and elevated office blood pressure readings; ambulatory blood pressure monitoring had been requested to clarify whether these represented sustained hypertension or a white-coat effect, but had not yet been performed. The patient was a non-smoker, with no regular alcohol consumption and no regular physical exercise. No known drug allergies were present.

Clinical presentation

The patient presented to a walk-in appointment at the USF Terras de Ferreira in early December 2025, with a 15-day history of left-sided hemicranial headache of a pressure-like quality, preceded by ipsilateral neck pain without identifiable trauma. In retrospect, the patient reported an episode of intense emotional stress (a family argument) during the weekend immediately prior to symptom onset, although she had not associated this with her complaint at presentation. The headache was progressively accompanied by left ptosis, ipsilateral scalp hyperesthesia, and taste disturbance affecting the posterior third of the tongue. She denied motor deficits, aphasia, visual changes, or diplopia.

On the basis of these findings, the general practitioner conducted a brief clinical assessment, including neurological examination, and referred the patient to the Emergency Department (ED) of Hospital Padre Américo, ULS Tâmega e Sousa, with clinical suspicion of cerebrovascular pathology.

Emergency department assessment

On ED admission, the patient was alert, cooperative, and fully oriented. Neurological examination revealed isochoric and photoreactive pupils, intact visual fields and ocular motility, left ptosis, left facial hypoesthesia, preserved motor strength, normal gait, and no dysmetria. Blood pressure was 168/112 mmHg, and heart rate was 83 bpm. Cardiopulmonary auscultation was unremarkable.

Initial investigations included full blood count, coagulation screen, renal function, electrolytes, and liver enzymes, all within normal limits. ECG showed sinus rhythm at 63 bpm. CT angiography of the head and neck was performed, revealing no intracranial hemorrhage or acute ischemic lesions, patent intracranial vessels, and narrowing of the left ICA at the post-bulbar segment, raising the suspicion of dissection (Figure 1). No occlusions or endoluminal thrombi were identified in the major intracranial arteries. Paranasal sinusitis (maxillary and ethmoidal) was an incidental finding.

**Figure 1:**
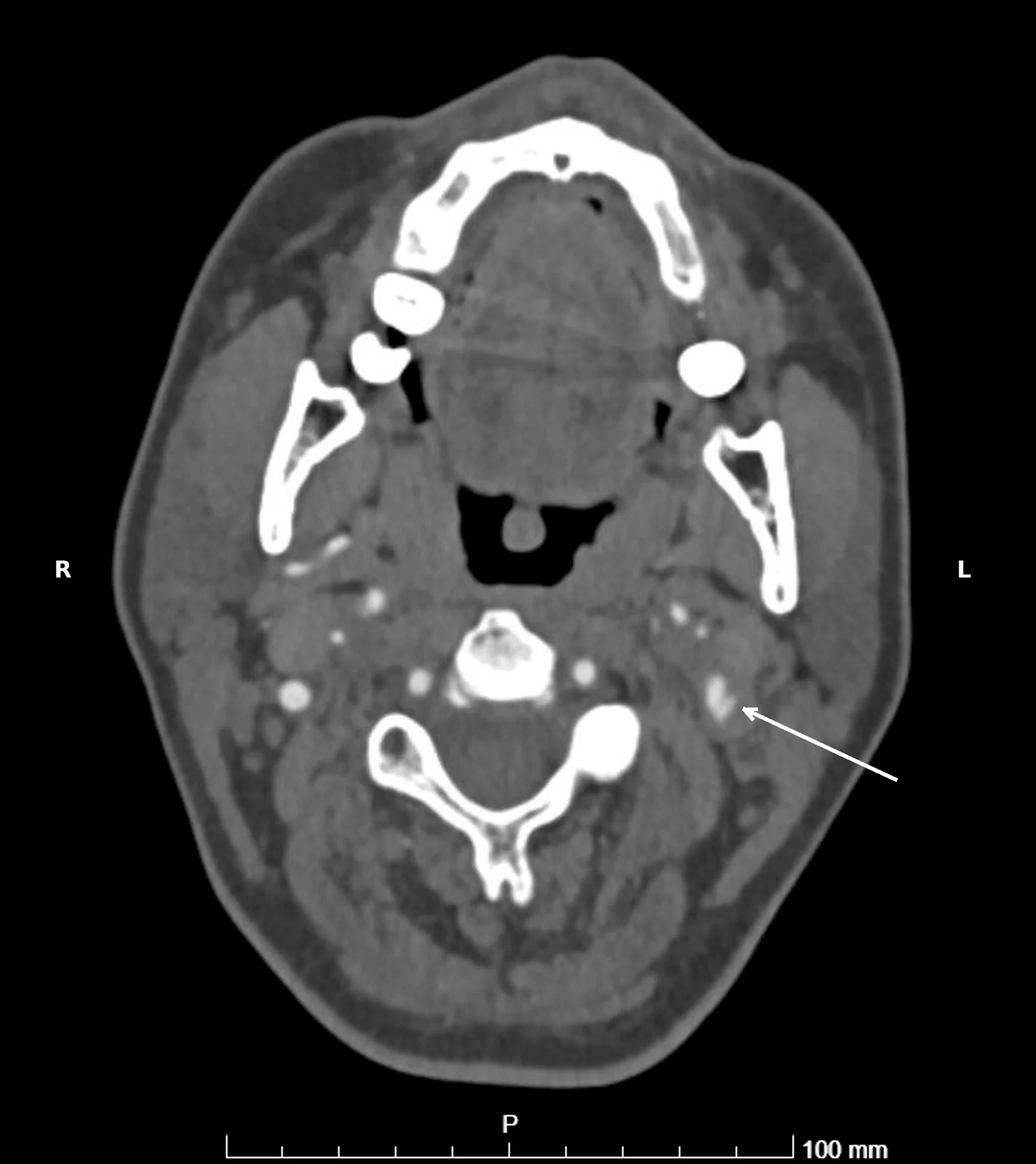
CT angiography showing reduced caliber of the left internal carotid artery. Axial CT angiography of the neck performed at presentation. The white arrow indicates the left internal carotid artery, showing reduced caliber compared with the normal contralateral vessel, consistent with arterial dissection. R: right; L: left.

Imaging was reviewed by the Neuroradiology Department at ULS São João, which concurred with the diagnosis of probable left ICA dissection and recommended medical treatment and complementary investigation. The patient was admitted to the stroke unit and commenced on dual antiplatelet therapy.

Inpatient workup and management

The patient was hospitalized for seven days. During this period, headache improved progressively with a gradual reduction in analgesic requirements. No new neurological deficits occurred, and facial hypoesthesia resolved completely.

The extended etiological workup revealed the following: erythrocyte sedimentation rate = 18 mm/h; glycosylated hemoglobin = 4.7%; lipid profile (total cholesterol = 184 mg/dL, high-density lipoprotein cholesterol = 61 mg/dL, low-density lipoprotein cholesterol = 102 mg/dL, triglycerides = 105 mg/dL); normal thyroid function (thyroid-stimulating hormone = 1.88 microIU/mL); folate = 5.0 ng/mL; vitamin B12 = 226 pg/mL; unremarkable serum protein electrophoresis. Immunological screening was negative for antinuclear antibodies, ANCA-PR3, ANCA-MPO, lupus anticoagulant, anti-beta-2-glycoprotein I IgG/IgM antibodies, and anticardiolipin IgG/IgM antibodies. Complement levels and immunoglobulin levels were normal, as was the homocysteine level. Serological tests were negative for anti-Treponema pallidum antibodies, and HIV and HCV tests were non-reactive. Angiotensin-converting enzyme level was pending at discharge.

Cervical Doppler ultrasound on hospital day 2 confirmed dissection of the left ICA at the post-bulbar segment, with a minimally opacified false lumen, moderate luminal stenosis, and no other significant findings. Transcranial Doppler showed no significant hemodynamic changes at the cranial base arteries.

Magnetic resonance (MR) angiography of the head and neck on hospital day 2, performed using a three-dimensional time-of-flight sequence, demonstrated no intracranial hemorrhagic or recent ischemic lesions; a small non-specific subcortical glial focus in the left posterior frontal lobe; patent dural venous sinuses without thrombosis; and reduction in caliber of the left ICA consistent with dissection. Repeat transcranial Doppler on hospital day 7 confirmed persistence of left ICA dissection findings with mild-to-moderate stenosis.

The case was reviewed by the Neurology Department at ULS Tâmega e Sousa, which confirmed the indication for continued dual antiplatelet therapy.

At discharge on hospital day 7, the patient was neurologically intact (National Institutes of Health Stroke Scale = 0; modified Rankin Scale = 0). Prescribed medications were as follows: aspirin 100 mg plus clopidogrel 75 mg (dual antiplatelet therapy, not to be discontinued without medical guidance); enalapril 5 mg (newly diagnosed hypertension); pantoprazole 20 mg (gastroprotection). Prior medications were maintained: topiramate 50 mg twice daily, desogestrel oral contraceptive, and zolmitriptan as needed. Discharge instructions included avoidance of strenuous activity, heavy lifting, cervical massage, and sudden neck movements; ambulatory blood pressure monitoring; and immediate ED presentation in the event of new neurological symptoms. The patient was referred for a neurology outpatient appointment within six weeks, with a repeat cervical vascular ultrasound.

Follow-up

At the neurology outpatient appointment six weeks after discharge, the patient reported persistent left hemicranial headache with partial response to paracetamol, along with significant scalp hyperalgesia and cervicalgia aggravated by neck movements. Contrast-enhanced ultrasound (CEUS) of the cervical vessels performed at the same visit identified a linear image consistent with an intimo-medial flap at the bulbar/post-bulbar transition of the left ICA, with contrast penetration into the false lumen, planimetric stenosis of 40-50% (without hemodynamic significance; peak systolic velocity of 88/40 cm/s), and multiple parietal irregularities compatible with vascular remodeling. Gabapentin 100 mg three times daily was initiated for neuropathic pain. Dual antiplatelet therapy was maintained, and a follow-up MR angiography was requested.

MR angiography of the head and neck 10 weeks after discharge showed no ischemic lesions, confirming reduction in caliber and parietal irregularity of the proximal left ICA with a lateral wall dilatation/pseudoaneurysm measuring approximately 5.4 mm (Figure 2), with recovery of caliber and flow beyond this level. No intracranial aneurysms or vascular malformations were identified.

**Figure 2:**
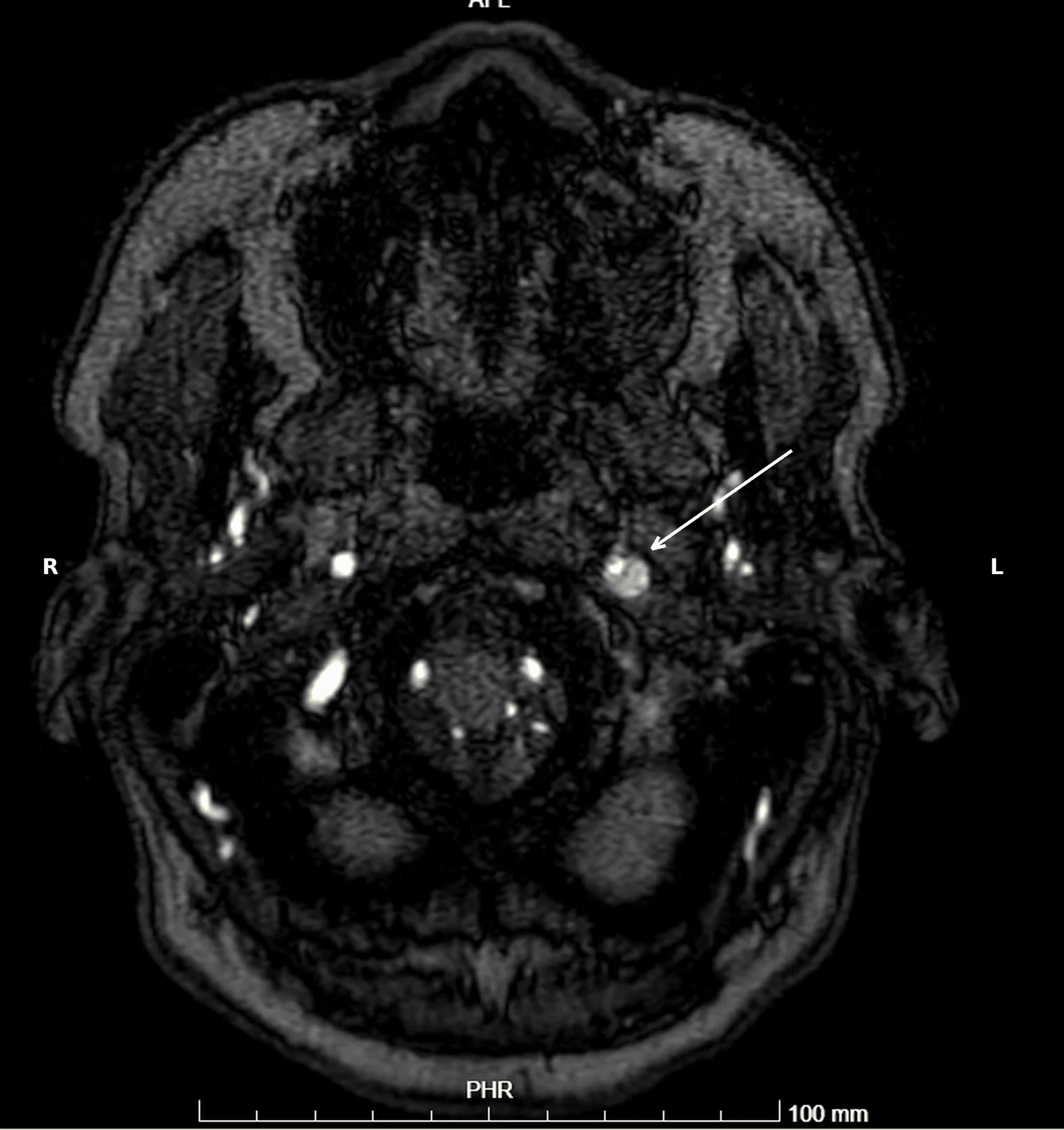
Magnetic resonance angiography showing pseudoaneurysm of the left internal carotid artery. Axial time-of-flight magnetic resonance angiography. The white arrow indicates the pseudoaneurysm arising from the left internal carotid artery, measuring approximately 5.4 mm. R: right; L: left.

Cervical vascular Doppler ultrasound four months after discharge confirmed findings consistent with left ICA dissection, with apparent pseudoaneurysm formation at the post-bulbar segment (approximately 5.5 mm) and signs of vascular remodeling, without evidence of hemodynamically significant stenosis.

The patient remains under regular neurology follow-up at ULS Tâmega e Sousa and continues to attend the USF Terras de Ferreira for cardiovascular risk factor management, blood pressure monitoring, continuity of care, and support regarding diagnosis-related concerns. The full clinical course is summarized in Table 1.

**Table 1:** Clinical timeline from symptom onset to most recent follow-up, spanning five months. BP: blood pressure; CEUS: contrast-enhanced ultrasound; CT-A: CT angiography; ICA: internal carotid artery; MRA: magnetic resonance angiography; mRS: modified Rankin Scale; NIHSS: National Institutes of Health Stroke Scale.

Timeline	Event/Findings	Action/Treatment
2 weeks before presentation	Progressive left-sided neck pain; no identifiable trauma or trigger	No medical contact at this stage
Day 1 (presentation)	Left hemicranial headache (15 days); left ptosis; scalp hyperesthesia; taste disturbance (posterior third of tongue)	Neurological examination performed; urgent referral to the emergency department
Hospital days 1-2	BP = 168/112 mmHg; CT-A: left ICA narrowing (post-bulbar); Doppler: false lumen, moderate stenosis; MRA: dissection confirmed, no ischemia	Admitted to the stroke unit; aspirin 100 mg + clopidogrel 75 mg; enalapril 5 mg; extended etiological workup (negative)
Hospital day 7 (discharge)	Neurologically intact; NIHSS = 0; mRS = 0	Discharged on hospital day 7; dual antiplatelet therapy maintained; neurology follow-up in 6 weeks
6 weeks after discharge	Persistent headache and cervicalgia; scalp hyperalgesia; CEUS: intimo-medial flap, 40–50% stenosis	Gabapentin 100 mg three times daily initiated; dual antiplatelet therapy continued; MRA requested
10 weeks after discharge	MRA: pseudoaneurysm ~5.4 mm, left ICA post-bulbar segment; no ischemia; normal distal flow	Conservative management; imaging surveillance continued
4 months after discharge	Doppler: pseudoaneurysm stable ~5.5 mm; vascular remodeling; no hemodynamically significant stenosis	Dual antiplatelet therapy maintained; joint primary care and neurology follow-up

## Discussion

This case illustrates the diagnostic challenge posed by spontaneous ICA dissection presenting without ischemic deficits, and highlights several features that are individually uncommon: the absence of motor or language involvement, glossopharyngeal nerve dysfunction manifesting as dysgeusia, and the subsequent development of a pseudoaneurysm during follow-up.

Spontaneous ICA dissection occurs most frequently between the third and fifth decades of life, often in individuals without classical cerebrovascular risk factors [1]. In this patient, no established predisposing factor was identified. The extended etiological workup, including screening for connective tissue and inflammatory disease, thrombophilia, and infectious causes, was negative, and there was no family history of arterial dissection or connective tissue disorder. Blood pressure was elevated at presentation (168/112 mmHg). Primary care records documented previous high-normal office readings, and ambulatory blood pressure monitoring had been requested to distinguish sustained hypertension from a white-coat effect but had not yet been performed at the time of the dissection. A formal diagnosis of hypertension had therefore not been established beforehand, and the contribution of blood pressure to the dissection cannot be determined in this case; antihypertensive treatment was started during the index admission. Similarly, although the patient was taking a desogestrel-only oral contraceptive, progestogen-only preparations carry a more favorable thrombotic risk profile than combined formulations, and we are not aware of evidence specifically linking them to cervical artery dissection; this exposure is therefore reported as a contextual finding rather than a contributory factor.

The patient retrospectively reported an episode of intense emotional stress during the weekend preceding symptom onset. No direct cervical trauma or forceful neck movement was reported at any point. This association is temporal only, and no mechanistic inference can be drawn from a single case; it is reported for completeness rather than as an established trigger.

The clinical presentation of this case illustrates the diversity of possible manifestations. The classic triad of neck and cranial pain, Horner syndrome, and cerebral ischemia is rarely complete [1]. In this patient, the absence of motor deficits or aphasia, manifestations that typically prompt urgent medical attention, alongside the presence of apparently subtle signs (ptosis, scalp hyperesthesia, taste disturbance) exemplifies how this condition may easily be misattributed to more common diagnoses such as migraine with atypical aura, trigeminal neuralgia, or otitis. The clinical findings observed in this patient, grouped by the anatomical structure involved, are presented in Table 2.

**Table 2:** Clinical manifestations by anatomical structure. Clinical findings grouped by the anatomical structure involved. All manifestations were left-sided and ipsilateral to the dissection. CN: cranial nerve.

Vascular	Oculosympathetic	Cranial nerves/sensory
Hemicranial headache	Left ptosis	CN IX: dysgeusia (posterior third of tongue)
Cervicalgia		CN V: facial hypoesthesia (resolved in-hospital)
Luminal stenosis 40-50%		Scalp hyperesthesia
No cerebral ischemia		

It is worth noting that ptosis was the only oculosympathetic sign documented. Pupils were isochoric and photoreactive, and neither miosis nor anisocoria was recorded; the finding is therefore described as isolated ipsilateral ptosis rather than Horner syndrome.

Taste disturbance affecting the posterior third of the tongue, reflecting involvement of the glossopharyngeal nerve (cranial nerve IX), is an uncommon but documented finding in ICA dissection, resulting from mechanical compression of lower cranial nerves by the expanding arterial wall hematoma at the level of the parapharyngeal space [4,7]. Its presence, together with ptosis and facial hypoesthesia, indicates involvement of structures anatomically adjacent to the cervical ICA and carries localizing diagnostic value.

From an imaging standpoint, CT angiography served as the first-line investigation in the ED, identifying the reduction in left ICA caliber and raising the hypothesis of dissection. MR angiography using a time-of-flight sequence demonstrated the luminal abnormality and, together with cervical Doppler ultrasound, supported the diagnosis. Detection of intramural hematoma depends substantially on the specific sequences acquired, in particular vessel-wall or fat-suppressed T1-weighted imaging, rather than on MR angiography alone. In this patient, the diagnosis rested on concordant luminal findings across CT angiography, cervical Doppler ultrasound, and MR angiography. Cervical Doppler ultrasound, widely available, also proved useful both in characterizing the stenosis and in longitudinal follow-up.

Antithrombotic therapy is standard in cervical artery dissection, and the CADISS trial found no significant difference in stroke recurrence between antiplatelet and anticoagulant strategies [8]. That trial did not, however, establish dual antiplatelet therapy as the preferred antiplatelet regimen for uncomplicated dissection. In this patient, the treating team elected to use aspirin plus clopidogrel in the context of an acute dissection with a patent but stenosed lumen and no established infarct, a decision made on clinical grounds rather than on the basis of comparative trial evidence for dual versus single antiplatelet therapy. The development of a pseudoaneurysm on follow-up imaging, measuring approximately 5.4 mm, represents a recognized complication of arterial dissection. Evidence from CADISS and related systematic reviews suggests that dissecting pseudoaneurysms have a generally benign natural history when managed conservatively, with the majority remaining stable or resolving spontaneously; however, ongoing imaging surveillance and specialist review are warranted [9,10].

The persistence of headache and cervicalgia beyond the acute phase, necessitating the introduction of gabapentin, is clinically relevant. Follow-up studies document that a significant proportion of patients with cervical artery dissection experience chronic pain after the acute event, with considerable impact on quality of life and functional capacity [5,11]. In this patient, the functional and occupational impact was substantial, with sustained work incapacity persisting throughout the follow-up period described.

From a general practice perspective, this case underlines the importance of not defaulting to a migrainous etiology in a patient with a known history of migraine when faced with headache of new character, a diagnostic pitfall sometimes termed the convenience exclusion diagnosis. The presence of new-onset symptoms (ptosis, hypoesthesia, taste disturbance), subacute evolution, and preceding neck pain are red flags that justify targeted etiological investigation [1]. This patient's profile corresponded closely to the factors independently associated with delayed recognition of cervical artery dissection: a documented history of migraine, headache as the dominant presenting symptom, and the absence of focal neurologic deficits [6].

## Conclusions

Spontaneous ICA dissection may present insidiously, with symptoms easily attributable to more common diagnoses. Early recognition of this entity, only possible with a high index of clinical suspicion, is important in preventing potentially devastating ischemic complications.

This case illustrates the role of general practice in the assessment of patients presenting with new or atypically characterized headache. The recognition of subtle cranial signs, the decision not to attribute the complaint to migraine without further investigation, and timely referral to the ED facilitated early diagnosis and treatment, and may have contributed to this patient's favorable clinical course. The follow-up course also underscores the complexity of this condition: pseudoaneurysm development, persistent chronic pain, and prolonged functional impairment reinforce the need for an integrated approach with effective articulation between primary care and specialist neurology services.

## Appendices

Patient perspective

The patient experienced the diagnosis with significant anxiety, centered primarily on concerns about recurrence and potential worsening of the dissection. The professional impact was substantial: as a photographer, she remained on medical leave at the time of this report, unable to resume her occupational activities. Clinical information provided throughout follow-up aimed to address her concerns and promote therapeutic adherence.
